# Nutritional Strategies and Dietary Patterns in Ménière’s Disease and Tinnitus: A Scoping Review of the Available Evidence

**DOI:** 10.3390/nu18132102

**Published:** 2026-06-27

**Authors:** Michał Klimas, Dominik Jucha, Sabina Krupa-Nurcek

**Affiliations:** 1 Faculty of Medicine, Collegium Medicum, University of Rzeszów, 35-310 Rzeszów, Poland; michal.klimas02@gmail.com (M.K.); dominik.jucha125@gmail.com (D.J.); 2 Department of Surgery, Faculty of Medicine, Collegium Medicum, University of Rzeszów, 35-310 Rzeszów, Poland

**Keywords:** Ménière disease, tinnitus, diet, dietary patterns, nutritional status, anti-inflammatory diet

## Abstract

Background: Ménière’s disease (MD) and tinnitus are common otological conditions that substantially impair quality of life. Although their pathophysiology remains incompletely understood, nutritional factors have been proposed to influence inner-ear microcirculation, water–electrolyte balance, oxidative stress and metabolic regulation. The objective of this scoping review was to comprehensively map the extent, nature and characteristics of existing research on nutritional strategies and dietary patterns applied in Ménière’s disease and tinnitus, and to clarify the mechanisms and clinical outcomes reported across studies. Methods: The review followed the Joanna Briggs Institute methodology and PRISMA-ScR guidelines. A comprehensive search of PubMed, Scopus, Web of Science, EBSCO, Cochrane Library and Google Scholar (2–12 March 2026) was conducted using the Population–Concept–Context model. Eligible studies included full-text human research (observational or interventional) and reviews published in English that examined dietary strategies in MD or tinnitus. Results: Of 273 records identified, 13 studies met inclusion criteria (6 on MD, 7 on tinnitus). Reported interventions included sodium reduction, adequate hydration, caffeine and alcohol modification, glycaemic stabilization, weight reduction, anti-inflammatory dietary patterns, and Mediterranean or DASH-style diets. Findings suggest potential symptom improvement in some patients. Conclusions: Available evidence indicates that nutritional interventions may serve as a valuable adjunct in the management of MD and tinnitus; however, their effectiveness has not been conclusively demonstrated. Well-designed, adequately powered randomised trials are still required to establish precise, evidence-based clinical guidelines.

## 1. Introduction

Ménière’s disease (MD) and tinnitus are among the most common otological disorders, which significantly reduce the quality of life of patients and pose a significant clinical challenge. MD is characterized by recurrent episodes of dizziness, fluctuating hearing loss, a feeling of ear fullness, and tinnitus, while tinnitus can occur both as a part of the clinical picture of this disease and as a separate entity with a multifactorial etiology [1,2,3]. Despite intensive research, the pathophysiology of both diseases remains not fully understood, and the available therapeutic methods often bring only partial or short-term improvement. Therefore, there is a growing interest in non-pharmacological supportive strategies, including nutritional interventions, which can affect the mechanisms underlying symptoms.

In recent years, a growing body of data indicates that diet may play an important role in modulating the functioning of the inner ear, especially by influencing microcirculation, water–electrolyte balance, inflammatory activity, oxidative stress and glucose metabolism. These mechanisms remain largely hypothetical and are supported mainly by indirect or preliminary evidence; therefore, their clinical relevance should be interpreted with caution [4,5]. In MD, the key pathophysiological component is the impaired volume and pressure of the endolymph [6]. In turn, studies on tinnitus analyze the role of components that affect neurotransmission, endothelial function, and oxidative stress.

At the same time, a growing body of research suggests that unfavorable eating habits [7,8,9,10] may negatively influence key pathophysiological processes associated with MD and tinnitus, including blood pressure regulation, glycemic stabilization, inflammation, and endothelial function [11].

In the absence of previous comprehensive studies and the high methodological variability of research, it becomes reasonable to conduct a comprehensive review that will allow for a broad and systematic presentation of the current state of knowledge regarding nutritional strategies and dietary patterns used in the treatment of MD and tinnitus [12,13]. The objective of this scoping review was to comprehensively map the extent, nature and characteristics of existing research on nutritional strategies and dietary patterns applied in Ménière’s disease and tinnitus, and to clarify the mechanisms and clinical outcomes reported across studies. The following research question was formulated: What nutritional strategies and dietary patterns have been studied in people with MD and/or tinnitus?

To avoid over-linking these two disease entities, in this review we clearly distinguish between data on MD—where the key pathophysiological component is impaired endolymphatic volume and pressure—and data on tinnitus, which may appear either as a feature of the clinical presentation of MD or as a separate condition with a multifactorial etiology.

### Pathophysiological Differences Between MD and Tinnitus

Although MD and tinnitus can co-occur, they are distinct disease entities with different pathophysiology, which is crucial for the interpretation of data on nutritional interventions. In MD, the primary mechanism involves a disturbance of endolymphatic homeostasis—specifically, impaired endolymphatic volume and pressure—which leads to paroxysmal symptoms such as dizziness, fluctuating hearing loss, a feeling of aural fullness, and tinnitus. While tinnitus can occur as a part of MD, it also exists as a separate condition with a multifactorial etiology. In contrast to MD’s fluid dynamics, isolated tinnitus is driven by neurotransmission disorders, oxidative stress, endothelial dysfunction, and micronutrient deficiencies. Nevertheless, common features between the two conditions—such as alterations in microcirculation, inflammatory processes, and oxidative stress—are rooted in shared systemic metabolic mechanisms rather than a common primary etiology.

## 2. Materials and Methods

### 2.1. Study Design

This scoping review was conducted in accordance with the Joanna Briggs Institute (JBI) methodology [14] and reported following the PRISMA-ScR guidelines [15]. A scoping review approach was selected to comprehensively map the breadth, nature and characteristics of the available evidence on nutritional strategies and dietary patterns in Ménière’s disease and tinnitus, given the heterogeneity of study designs, outcomes and methodological quality in this field. In line with JBI guidance, a formal risk-of-bias assessment was not performed, as the objective of a scoping review is to explore and summarize existing evidence rather than evaluate the effectiveness of interventions. A protocol specifying the research question, PCC framework, eligibility criteria, search strategy and data-charting procedures was developed a priori, although it was not registered in an external repository [16].

### 2.2. Inclusion and Exclusion Criteria

To ensure a transparent and rigorous selection process, eligibility criteria were divided into publication-related and research-related specifications.

#### 2.2.1. Publication-Related Criteria

Studies were eligible for inclusion if they were peer-reviewed original research articles (both observational and experimental), systematic reviews, or narrative reviews. Publications had to be available in full text and published exclusively in the English language. Dissertations, conference abstracts, book chapters, case reports, commentaries, letters to the editor, and animal-testing or in vitro studies were excluded.

#### 2.2.2. Research-Related Criteria (PICOS Framework)

To enhance clarity and systematically map the evidence, the research-related eligibility criteria were structured using the PICOS framework as follows:

Population (P): patients diagnosed with Ménière’s disease (MD) or tinnitus, without restrictions regarding age, gender, or place of treatment.

Intervention (I): any nutritional strategy, dietary modification, or dietary pattern (e.g., sodium restriction, hydration strategies, glycaemic stabilization, anti-inflammatory diets, Mediterranean, or DASH dietary patterns, weight-reduction diets).

Comparison (C): any comparator, including usual care, no intervention, placebo, or alternative dietary/lifestyle approaches. Consistent with scoping review methodology, studies without a control or comparator group were also eligible.

Outcomes (O): any reported outcomes related to symptom severity, quality of life, metabolic or inflammatory markers, audiovestibular function or patient-reported symptom changes. No outcome-based exclusions were applied.

Study design (S): observational designs (cross-sectional, cohort, case–control) and experimental studies (both randomized and non-randomized interventional trials). The term “experimental studies” was adopted broadly to accurately reflect the inclusion of all interventional designs rather than exclusively randomized controlled trials.

### 2.3. Search Strategy

The authors searched the following databases: PubMed, Scopus, EBSCO, Web of Science, Google Scholar and Cochrane Library. The following keywords were used: “tinnitus”, “diet”, “diet in tinnitus”, “influence diet in tinnitus”, “MD”, “diet”, “influence diet in MD”, as well as their combinations combined with AND and OR operators. All found publications were pre-evaluated based on titles and abstracts to exclude papers unrelated to the subject matter of the review. Any discrepancies in the assessment were resolved through a joint discussion of the researchers until a full consensus was reached on the final set of articles. The search process started on 2 March 2026, and ended on 12 March 2026.

The selection of databases was guided by JBI recommendations for comprehensive evidence mapping and by the multidisciplinary nature of the topic, which spans otology, nutrition, internal medicine and lifestyle medicine. PubMed, Scopus and Web of Science were included as core biomedical and multidisciplinary databases, ensuring broad coverage of clinical and epidemiological studies. EBSCO (Medline Complete, CINAHL) was added to capture additional clinical, nursing and allied-health literature. The Cochrane Library was included to identify existing systematic reviews and interventional evidence. Google Scholar was used as a supplementary source to ensure retrieval of grey literature and studies not indexed in traditional databases. This combination maximizes sensitivity and minimizes the risk of missing relevant evidence.

The strategy was developed in accordance with PRISMA-ScR, the JBI Manual for Evidence Synthesis, and the principles of search transparency. The search results are presented in Table 1.

Although the PRISMA flow diagram presents two thematic pathways (Ménière’s disease and tinnitus), the literature search itself was conducted as a single, unified search using combined search strings that included terms for both conditions. The separation into MD-specific and tinnitus-specific records occurred only during the screening and data-charting phases, reflecting the two components of the research question rather than two independent searches.

### 2.4. Extraction of Data

A form developed in accordance with the JBI Scoping Review Guidelines [14] was used to compile the data, including key information from the analysed sources. The data extraction process—referred to in the scoping reviews as “data charting” [15,17]—was carried out independently by two reviewers. To identify the appropriate studies, the Population–Concept–Context (PCC) scheme was used. From each article, information such as the name of the first author, year of publication, country, results, and main conclusions was obtained. The authors carried out the entire process of compiling the data in Microsoft Excel.

### 2.5. Process for Including Publications in the Review

**Figure 1:**
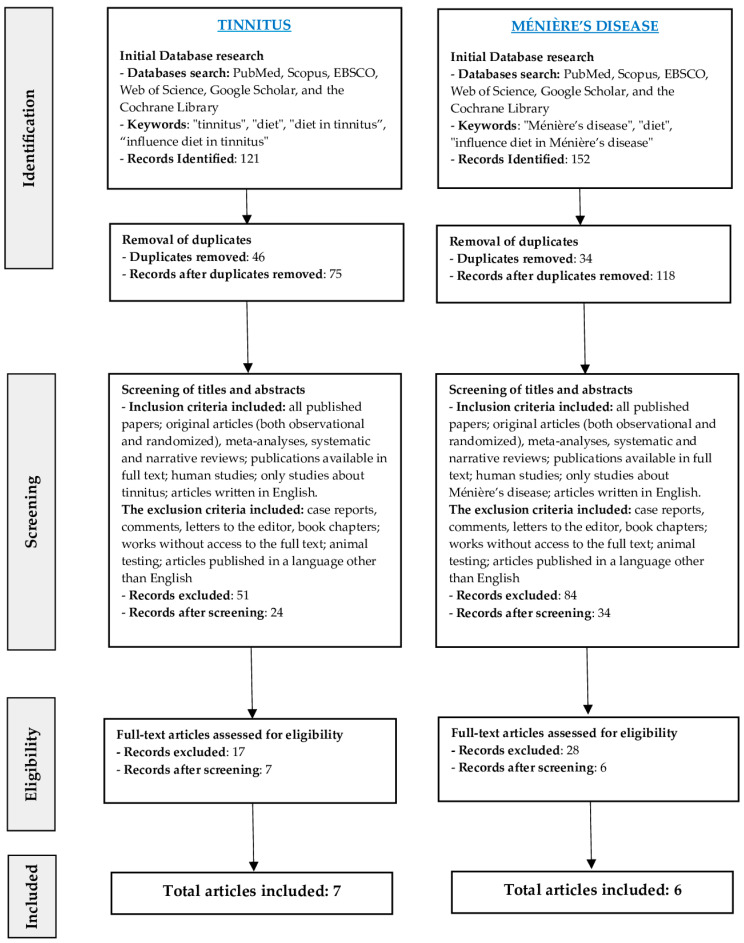
Literature search and selection flowchart for this review.

The study selection process was conducted by three reviewers. Two reviewers (MK and DJ) independently screened all titles and abstracts, followed by independent full-text assessment of potentially eligible studies. Any discrepancies at either stage were resolved through discussion. When consensus could not be reached, a third reviewer (SK.N) acted as an adjudicator. This procedure ensured independent assessment and minimized the risk of selection bias, in accordance with JBI and PRISMA-ScR recommendations. All articles were closely related to the topic of the scoping review (Figure 1). After removing duplicates (tinnitus n = 46, MD n = 34), 75 articles on tinnitus and 118 on MD remained. After reviewing the articles according to the inclusion and exclusion criteria (tinnitus n = 51, MD n = 84), 24 articles on tinnitus and 34 on MD remained. For tinnitus, 17 articles did not contain full text and were excluded. For MD, 28 articles were excluded at this stage. As a result, after meeting all requirements, 7 articles on tinnitus and 6 on MD were included in the review.

### 2.6. Justification for the Lack of a Formal Assessment of Methodological Quality

In the context of this review, which includes studies with high heterogeneity of designs, populations, interventions and outcome measures, the use of a single bias risk assessment tool would not be possible or methodologically justified. At the same time, limitations to the quality of the available studies—such as small samples, observational nature or subjective measurements of symptoms—have been taken into account when interpreting the results and discussed in detail in the Limitations section.

The protocol was not registered or published in an external repository. All methodological steps—including database selection, search strategy, study screening, and data charting—were performed systematically to ensure transparency and reproducibility.

## 3. Results

Our scoping review initially identified a total of 273 articles (121 on tinnitus and 152 on MD), of which 7 on tinnitus and 6 on MD were ultimately included in the analysis. The studies were conducted in the USA (n = 1), India (n = 1), Taiwan (n = 1), Germany (n = 1), Egypt (n = 1), Turkey (n = 3), China (n = 2), the UK (n = 2), and Canada (n = 1). The literature search and screening process is summarized in the PRISMA flowchart (Figure 1), and the detailed characteristics of the included studies are presented in Table 2 and Table 3.

The narrative synthesis presented below is strictly grounded in the evidence extracted from the 13 included studies. Several of the included publications were narrative or clinical reviews that did not report sample size, population characteristics, or other primary-study details. For this reason, some fields in Table 1 and Table 2 remain blank (“–”), reflecting the absence of such information in the original sources rather than incomplete data extraction. To enhance clarity, the text below explicitly distinguishes between primary studies, systematic reviews, narrative reviews and clinical reviews, and all thematic observations are directly linked to the data charted in the tables.

Although several dietary approaches—such as the Mediterranean diet, anti-inflammatory patterns, sodium reduction, glycemic stabilization or targeted micronutrient supplementation—have been proposed as potentially helpful, the overall strength of evidence identified in this review remains low. Findings across the reviewed studies are heterogeneous, often based on small samples, and frequently rely on subjective symptom reporting. As such, these interventions should be viewed as promising but unproven, and their clinical relevance remains to be established.

## 4. Dietary Strategies and Dietary Components in MD and Tinnitus

Interest in the role of diet in the management of tinnitus and MD is steadily increasing, due to both the limited effectiveness of available pharmacological therapies and growing patient awareness regarding lifestyle modifications. Both conditions possess complex, multifactorial etiologies involving metabolic, vascular, inflammatory, and fluid-dynamics pathways within the inner ear [22].

While numerous mechanistic pathways have been proposed to explain how nutrition modulates audiovestibular function, the current body of literature remains largely exploratory and fragmented. Many recommendations rely heavily on observational findings, biological plausibility, or small-scale trials, meaning that any potential clinical benefits must be interpreted with caution [23]. For scientific precision, data regarding MD and isolated tinnitus are discussed below with a clear pathophysiological distinction to avoid inappropriate conceptual simplification.

### 4.1. Sodium Intake and Fluid Regulation

Sodium restriction represents the classic and most frequently discussed dietary intervention for MD, rooted in historical frameworks aiming to stabilize endolymphatic volume and pressure. The underlying rationale assumes that excessive sodium intake promotes systemic fluid retention, thereby exacerbating endolymphatic hydrops.

In clinical practice, a multi-modal approach combining dietary salt restrictions, stress management, and pharmacotherapy is widely implemented to control vertigo episodes. For instance, interventional data have demonstrated that a low-sodium diet coupled with adequate water intake can improve hearing thresholds and relieve definitive vertigo symptoms in specific patient cohorts. However, systematic reviews within our sample highlight profound uncertainty, concluding that robust evidence from high-quality randomized controlled trials (RCTs) is still lacking to conclusively support or refute routine sodium restriction [24].

Importantly, this mechanism is exclusive to the fluid mechanics of MD. In the context of tinnitus, there is no empirical scientific backing to justify standard clinical recommendations for dietary salt restriction, and routine sodium limitations are not supported by tinnitus-specific studies.

### 4.2. Stimulants: Caffeine and Alcohol Modification

Caffeine and alcohol are routinely identified as potentially unfavorable dietary components capable of modulating inner-ear function. Caffeine, a potent central nervous stimulant, can augment neuronal excitability and alter microvascular blood flow. In clinical practice, some patients report a subjective increase in tinnitus perception following caffeine consumption [25]. Conversely, epidemiological data indicate that baseline daily consumption of caffeine may negatively correlate with tinnitus incidence in the general population, and abrupt caffeine withdrawal can temporarily worsen symptoms. Thus, evidence regarding routine caffeine restriction for tinnitus remains weak, and a gradual reduction rather than complete elimination is generally recommended. For MD, the direct clinical impact of caffeine is less clear-cut, though its reduction is often incorporated into general lifestyle modifications [22,26].

Alcohol consumption similarly interacts with inner-ear microcirculation and systemic electrolyte balance, potentially precipitating acute vertigo attacks in MD patients. While moderate consumption of specific beverages (e.g., red wine) is occasionally tied to broader cardiovascular benefits, observational data show highly inconsistent individual reactions. Consequently, the current consensus favors individual patient monitoring and the avoidance of alcohol only if a distinct exacerbation of symptoms is documented [27].

### 4.3. Carbohydrate Metabolism, Glycemic Index, and Caloric Restriction

The role of glucose-insulin metabolism and carbohydrate disorders is receiving increasing attention in neuro-otology. Diets characterized by a high glycemic index and excessive monosaccharide intake trigger rapid fluctuations in blood glucose and insulin levels. These metabolic spikes can disrupt inner-ear homeostasis through vascular endothelial stress, chronic low-grade inflammation, and shifts in the osmotic pressure of labyrinthine fluids. While stabilization of blood glucose levels via reduced simple sugar intake and increased dietary fiber theoretically mitigates these osmotic and metabolic shifts, current evidence supporting direct causality remains limited [28,29,30].

Interestingly, weight management and systemic metabolic interventions have emerged as particularly effective supportive strategies for chronic tinnitus. Clinical trials demonstrate that structured weight-loss programs utilizing calorie-restricted diets and physical activity significantly alleviate subjective tinnitus severity and enhance quality of life in obese cohorts. Furthermore, combining caloric restriction with aerobic exercise has been shown to simultaneously optimize metabolic syndrome components and mitigate chronic subjective tinnitus-related discomfort in elderly populations. These findings suggest that addressing systemic metabolic inflammation may yield superior clinical outcomes compared to isolating single dietary components.

### 4.4. Micronutrient Status and Targeted Supplementation

In the context of isolated tinnitus, the potential impact of specific dietary components such as trace elements and vitamins is heavily analyzed. Deficiencies in key elements—specifically vitamin B12, zinc, magnesium, and vitamin D—have been repeatedly associated with increased subjective symptom severity.

- B-Complex Vitamins (B6, B9, B12): Essential for homocysteine metabolism; elevated homocysteine levels induce endothelial dysfunction and microvascular stress, compromising blood supply to the inner ear. Supplementation may alleviate symptoms, particularly in patients with baseline metabolic disturbances.
- Magnesium: Participates directly in regulating neuronal excitability and vascular tone. Baseline deficiencies correlate with heightened susceptibility to tinnitus, and targeted supplementation has been shown to reduce subjective distress [25].
- Zinc: Possesses critical neuroprotective and antioxidant properties. While overall trial data are mixed, symptomatic improvement is primarily observed in individuals with confirmed baseline zinc deficiencies [29].

Crucially, routine supplementation of these components in patients without documented clinical deficiencies is entirely unsupported by empirical evidence [23,31]. Furthermore, literature in our sample indicates that these micronutrient associations apply exclusively to tinnitus; available studies on MD have failed to demonstrate analogous pathological links with trace element or vitamin deficiencies.

### 4.5. Systemic Dietary Patterns: Mediterranean, DASH, and Anti-Inflammatory Diets

Rather than isolating individual nutrients, contemporary research emphasizes the synergistic mechanisms of comprehensive dietary patterns. Both MD and tinnitus are hypothesized to involve underlying microvascular stress, localized inflammatory activity, and oxidative cellular damage.

The Mediterranean diet—abundant in antioxidants (vitamins C and E, carotenoids, polyphenols), omega-3 polyunsaturated fatty acids, and dietary fiber—exerts documented systemic anti-inflammatory and vascular effects [32,33,34,35]. Importantly, it should be noted that many key antioxidants found in the diet, such as polyphenols, are not formally classified as nutrients, further justifying a broader focus on dietary components. Mechanistically, these components neutralize free radicals capable of damaging cochlear hair cells and auditory neurons, while simultaneously optimizing endothelial integrity. Epidemiological population studies suggest that high adherence to Mediterranean-style or antioxidant-rich patterns correlates with lower tinnitus prevalence and severity, though rigorous interventional trials are still required to definitively verify clinical efficacy [31].

Similarly, the DASH (Dietary Approaches to Stop Hypertension) diet modulates audiovestibular symptoms through systemic vascular stabilization. In MD, the primary utility of the DASH pattern stems from its structural combination of low sodium and high potassium, magnesium, and calcium content, which collectively support systemic fluid balance and counteract fluctuations in endolymphatic pressure [30]. In tinnitus, the DASH framework operates by enhancing vascular endothelial barrier function and suppressing pro-inflammatory cytokine cascades [29,36,37]. Consequently, while data from large-scale prospective trials remain sparse, these broad dietary strategies are safe, exceptionally well-tolerated, and serve as valuable non-pharmacological adjuncts to standard clinical care [38,39,40,41]. The proposed pathophysiological mechanisms, dietary sources, and targeted clinical implications of the specific dietary components and systemic patterns discussed throughout this section are summarized in Table 4.

## 5. Discussion

The primary objective of this scoping review was to comprehensively map the extent, nature, and characteristics of the existing literature regarding nutritional strategies and dietary patterns in Ménière’s disease (MD) and tinnitus. By evaluating 13 included studies (6 focusing on MD and 7 on tinnitus), this work highlights a growing academic and clinical interest in non-pharmacological supportive approaches, driven largely by the suboptimal efficacy of conventional medical therapies. However, our synthesis underscores a critical disparity between widespread, traditional clinical recommendations and the actual strength of the empirical evidence supporting them. As reviewed by Shim et al., this approach stems from historical frameworks aiming to stabilize endolymphatic volume and pressure [20]. In our review, some support for this strategy was observed; for instance, Yang et al. demonstrated that a low-sodium diet combined with adequate water intake significantly improved hearing thresholds while relieving dizziness and tinnitus in a cohort of 50 MD patients [9]. Furthermore, Wu et al. reinforced that vertigo episodes could be successfully managed through a multi-modal approach combining salt restriction, stress reduction, and pharmacotherapy [18]. Despite these positive observations, the broader literature demands substantial caution. Systematic and clinical reviews within our sample reveal profound uncertainty. Webster et al. evaluated lifestyle and dietary interventions against placebo or no treatment, concluding that the available evidence is of low to very low certainty, making definitive clinical conclusions impossible [5]. This is directly echoed by Hussain et al., who stated that no high-quality evidence from randomized controlled trials exists to conclusively support or refute the restriction of salt, caffeine, or alcohol in MD patients [19]. Similarly, Oğuz et al. noted that while eating habits clearly modulate symptoms by interacting with fluid metabolism and inflammatory pathways, the precise clinical effectiveness of routine dietary restrictions remains unproven [10]. These contradictions suggest that while sodium restriction may benefit a subset of patients, its universal application without considering individual metabolic or fluid-balance variations lacks a robust empirical foundation.

In the context of tinnitus, the therapeutic focus shifts from fluid mechanics to neuroprotection, vascular endothelial integrity, and the mitigation of oxidative stress. Wadhwa et al. emphasized the therapeutic potential of patient empowerment, where adopting healthy dietary habits, optimizing sleep hygiene, and managing stress can alleviate subjective symptom severity and improve overall quality of life [6]. The structural composition of the diet appears to influence these outcomes. Chen et al. indicated that high-fat diets may exacerbate audio-vestibular dysfunction via oxidative stress pathways, whereas high carbohydrate and simple sugar intakes correlate positively with symptom prevalence [7]. Conversely, they highlighted the potential protective effects of antioxidant vitamins (A, C, and E) and Mediterranean-style dietary patterns. This is partially corroborated by Zhang et al., whose epidemiological data demonstrated that higher daily consumption of fruit, dietary fiber, dairy products, and even caffeine was negatively correlated with tinnitus incidence [13].

Interestingly, metabolic and weight management strategies emerged as particularly effective interventions for chronic tinnitus. In our review, primary studies by Özbey-Yücel et al. and Özbey-Yücel et al. consistently demonstrated that weight loss achieved through targeted nutritional programs and physical activity successfully alleviated tinnitus severity and boosted quality of life in obese cohorts [12,21]. Furthermore, Ismail et al. confirmed that a 12-week intervention combining a calorie-restricted diet with treadmill training yielded significant improvements in both metabolic syndrome components and chronic subjective tinnitus-related discomfort among elderly individuals [11]. These findings suggest that addressing systemic metabolic inflammation and vascular health may be more effective for managing tinnitus than isolating single nutrients. Nevertheless, the field of tinnitus research faces identical methodological hurdles to those seen in MD. Hofmeister et al. conducted a rigorous review of diet quality and supplement efficacy, reporting that the evidence connecting overall diet quality to tinnitus remains very weak [8]. Critically, they concluded that routine dietary supplements are ineffective and should not be recommended by clinicians in the absence of a documented deficiency, while also finding no empirical scientific backing for the standard clinical practice of restricting caffeine and dietary salt in tinnitus patients.

Historically, literature in this domain has frequently conflated the two conditions due to their frequent clinical co-occurrence. However, as outlined in our pathophysiological framework, MD is inherently a disorder of endolymphatic pressure, whereas tinnitus represents a multifactorial neuro-otological symptom tied to neurotransmission anomalies and microvascular stress. Blending these entities results in conceptual simplification and risks generating inappropriate clinical guidelines.

## 6. Conclusions

Available evidence suggests that certain dietary strategies may offer supportive benefits for some individuals with MD or tinnitus; however, the current body of literature is weak, inconsistent, and insufficient to draw definitive conclusions. Nutritional interventions should therefore be considered adjunctive rather than evidence-based treatments. High-quality experimental studies are needed to determine whether these dietary approaches have clinically meaningful effects. The most commonly reported interventions include reducing sodium, caffeine and alcohol intake, stabilising glucose-insulin metabolism, using anti-inflammatory diets and supplementing deficiencies in selected nutrients such as magnesium, B vitamins, zinc and omega-3 fatty acids. Dietary patterns with documented beneficial effects on metabolic and vascular health—in particular the Mediterranean diet and the DASH diet—can support microcirculation, reduce oxidative stress and modulate inflammation, which theoretically helps to alleviate the symptoms of both conditions. At the same time, clinical observations and study results suggest that individual patient responses to nutritional interventions may vary, with the effectiveness of individual recommendations depending on concomitant metabolic disorders, lifestyle and overall health. Despite the growing interest in nutrition in otological diseases, the scientific literature remains scattered and heterogeneous, with many recommendations based on observational studies or small clinical trials. However, available data indicate that nutritional interventions are safe and well-tolerated and can be a valuable complement to pharmacological and non-pharmacological therapies. Further, well-designed randomised trials are needed to conclusively assess the effectiveness of different dietary strategies, identify their mechanisms of action, and develop precise, evidence-based clinical guidelines for patients with MD and tinnitus.

At the same time, the conclusions of this scoping review should be interpreted with caution. Although nutritional strategies appear safe, feasible, and potentially supportive as adjuncts to standard care, the current evidence base is insufficient to formulate strong or universal clinical recommendations. The available studies are heterogeneous, often small, and generally of low methodological certainty, which limits the strength of inferences that can be drawn. Therefore, these dietary approaches should be regarded as promising but still exploratory options, whose effectiveness remains uncertain and requires confirmation in well-designed, adequately powered prospective studies.

## 7. Limitations

This scoping review has several important limitations that need to be taken into account when interpreting the results. First, according to the Joanna Briggs Institute methodology and the PRISMA-ScR guidelines, no formal assessment of methodological quality or risk of bias was conducted in the included studies. Although such an assessment is not required in a scoping review, its absence limits the ability to assess the reliability and robustness of the available evidence. This is particularly important when many of the studies included are observational, involve small groups of participants, or are based on subjective measures of symptom severity, which increases the risk of bias and makes it difficult to draw clear conclusions.

The included studies were characterised by significant heterogeneity in terms of research designs, population characteristics, nutritional interventions used and how results were evaluated. This variability has made it impossible to compare studies directly and has limited the ability to identify consistent patterns of effectiveness of different nutritional strategies.

The evidence base in the field of nutrition in the context of MD and tinnitus remains fragmented and often exploratory, with a predominance of cross-sectional studies, observational analyses and narrative clinical descriptions. Therefore, the results of this review should be considered as a synthetic representation of the current state of knowledge and not as a basis for formulating clinical recommendations. Future studies—especially well-designed randomised controlled trials and prospective cohort studies—should include standardised outcome measures and allow for a formal assessment of the risk of bias, allowing for a more precise determination of the role of nutritional interventions in the treatment of these conditions.

Another limitation of this review is the relatively limited breadth and depth of literature analysis. Due to the inclusion criteria adopted based on the PCC model, only studies in which nutritional interventions or dietary patterns were directly assessed in the context of tinnitus or MD were included. This approach, while consistent with the scoping review methodology, narrowed down the number of eligible publications and did not allow for a full reflection of the complexity of the relationship between nutrition and the pathophysiological mechanisms of these diseases. As a result, many important mechanistic studies on inflammatory processes, oxidative stress, metabolic dysfunction, endothelial function, gut microbiota, or neurovascular mechanisms, which may be relevant to the interpretation of the role of diet but did not meet the criterion of direct dietary intervention, were not included. In addition, some of the cited works are older in nature and have limited clinical relevance, although they have been included due to their historical significance and impact on current practices. In future studies, it will be necessary to expand the scope of the search to include newer, more advanced clinical and mechanistic studies in order to obtain a more complete and comprehensive picture of the available evidence.

A further limitation concerns the mechanistic explanations discussed in this review. Many proposed pathways—such as inflammation, oxidative stress, endothelial dysfunction, microbiota alterations or neurotransmission changes—are supported mainly by preliminary or indirect evidence. As a scoping review, we aimed to map these hypotheses as they appear in the literature; however, they should not be interpreted as established causal mechanisms. Future mechanistic and interventional studies are required to clarify their clinical relevance.

This review represents the first attempt to comprehensively and systematically organize the dispersed literature on nutritional strategies and dietary patterns in MD and tinnitus, with a clear separation of the two disease entities according to their different pathophysiology. Its strengths are the use of JBI and PRISMA-ScR methodologies, a broad search covering six main databases, and a synthetic comparison of dietary interventions with potential biological mechanisms, which has not been presented in existing reviews before. The review also identifies key research gaps, pinpointing areas that require well-designed interventional studies. The limitations are mainly due to the quality of the available data: most studies are characterised by small samples, methodological heterogeneity, subjective measures of symptom severity, and a lack of high-power randomised trials, which limits the possibility of drawing unambiguous clinical conclusions. In addition, due to the nature of the scoping review, no formal bias risk assessment has been conducted, which is in line with the JBI guidelines, but may affect the interpretation of the strength of evidence.

In addition, several methodological constraints should be acknowledged. Although detailed search strings were provided for each database, the reproducibility of the search strategy may be limited due to differences in indexing across platforms and the use of simplified queries in Google Scholar. Furthermore, in line with JBI and PRISMA-ScR methodology, no formal critical appraisal of the included studies was performed; while appropriate for a scoping review, this limits the ability to assess the robustness and internal validity of the evidence base. The inclusion of both primary studies and review articles may also have resulted in partial overlap of data, which is an inherent limitation of evidence-mapping approaches. Finally, restricting the search to English-language, full-text publications may have introduced language and selection bias, potentially leading to the omission of relevant studies published in other languages or available only as abstracts.

## Figures and Tables

**Table 1:** Summary of Search Strategy.

Database	Search String (Core Structure)	Filters Applied	Records Retrieved	Records After Deduplication
PubMed	(“Tinnitus”[MeSH] OR tinnitus[tiab] OR “subjective tinnitus”[tiab]) AND (“Meniere Disease”[MeSH] OR meniere[tiab] OR “MD”[tiab]) AND (“Diet”[MeSH] OR diet[tiab] OR “diet therapy”[MeSH] OR “dietary patterns”[tiab] OR “Mediterranean diet”[tiab] OR “DASH”[tiab] OR “anti-inflammatory diet”[tiab] OR micronutrient[tiab] OR “sodium, dietary”[MeSH] OR caffeine[MeSH] OR alcohol[tiab] OR “glycemic index”[MeSH])	Humans; English; Full text	112	86
Scopus	TITLE-ABS-KEY(tinnitus OR “subjective tinnitus”) AND TITLE-ABS-KEY(“Meniere disease” OR “Ménière”) AND TITLE-ABS-KEY(diet OR “dietary pattern” OR “Mediterranean diet” OR DASH OR “anti-inflammatory diet” OR micronutrient OR sodium OR caffeine OR alcohol)	English; Article; Review	98	74
Web of Science Core Collection	TS = (tinnitus OR “subjective tinnitus”) AND TS = (“Meniere disease” OR “Ménière”) AND TS = (diet OR “diet therapy” OR “dietary pattern” OR “Mediterranean diet” OR DASH OR “anti-inflammatory diet”)	English; Article; Review	61	47
EBSCO (Medline Complete, CINAHL)	(MH “Tinnitus” OR tinnitus OR “subjective tinnitus”) AND (MH “Meniere Disease” OR meniere OR “Ménière”) AND (MH “Diet” OR diet OR “diet therapy” OR “dietary pattern” OR “Mediterranean diet” OR DASH OR “anti-inflammatory diet”)	Humans; English; Full text	54	39
Cochrane Library	(tinnitus:ti,ab,kw OR “subjective tinnitus”:ti,ab,kw) AND (“Meniere disease”:ti,ab,kw OR “Ménière”:ti,ab,kw) AND (diet:ti,ab,kw OR “dietary pattern”:ti,ab,kw OR “Mediterranean diet”:ti,ab,kw OR DASH:ti,ab,kw)	No filters	12	10
Google Scholar	“tinnitus” AND “diet” AND (“Meniere” OR “Ménière”) AND (“Mediterranean diet” OR “DASH” OR “anti-inflammatory diet”)	First 200 results screened	200 screened	17 included

**Table 2:** Characteristics and findings of studies included in this review about nutritional strategies and dietary patterns used to treat MD.

Author, Year	Country	Aim of the Study	Participants	Results and Findings
Webster KE. et al., 2023 [5]	UK	Assessing the benefits and harms of lifestyle and dietary interventions compared to placebo or no treatment in people with MD.	-	√ The evidence that lifestyle or dietary interventions have an impact on MD is very uncertain √ Only two studies comparing a diet or lifestyle intervention with placebo or no treatment have been defined
Yang X. et al., 2024 [9]	China	Investigating the Effect of a Low-Sodium Diet with Adequate Water Intake on Clinical Efficacy in MD.	50 patients with MD	√ A low-sodium diet with adequate water intake improved hearing and relieved dizziness and tinnitus in MD patients
Oğuz E. et al., 2021 [10]	Turkey	To examine the relationship between MD and dietary intervention, which is often used in the prevention and treatment of MD attacks	-	√ Lifestyle changes, dietary modifications, diuretics, vasodilator corticosteroids, intraductal steroids, surgical methods are some of the treatments for MD √ The authors indicate that eating habits may affect the course and severity of symptoms of MD √ Diet can modulate symptoms by affecting fluid metabolism and inflammatory processes √ Dietary recommendations are used routinely, but their effectiveness is not certain
Wu V. et al., 2019 [18]	Canada	Presenting family doctors with the current approach to the diagnosis and treatment of MD, discussing its natural course and indicating how to start treatment before an ENT consultation	-	√ Symptoms of dizziness can be controlled through a combination of dietary salt restriction, stress reduction, and medical therapy (betazine, diuretics, or both)
Hussain K. et al., 2018 [19]	UK	Evaluation of the effects of dietary restriction on salt, caffeine and alcohol consumption in patients with MD or syndrome	-	√ There is no evidence from randomized controlled trials to support or refute the restriction of salt, caffeine, or alcohol intake in patients with MD or syndrome
Shim T. et al., 2020 [20]	USA	Determining the historical origins of the use of a salt-restricted diet as an intervention for MD	-	√ Salt-restricting diet remains the primary first-line treatment for MD √ Various publications have both supported and argued this treatment, and the evidence for its validity remains inconclusive

**Table 3:** Characteristics and findings of studies included in this review about nutritional strategies and dietary patterns used to treat tinnitus.

Author, Year	Country	Aim of the Study	Participants	Results and Findings
Wadhwa S. et al., 2024 [6]	India	To collect and summarize current knowledge about the impact of diet, hydration, physical activity, stress, sleep, and stimulants on the symptoms of tinnitus	-	√ Specific dietary patterns and lifestyle choices can affect the severity and frequency of tinnitus symptoms. √ People affected by tinnitus are encouraged to actively engage in care by adopting healthy eating habits, managing stress levels, prioritizing sleep hygiene, and incorporating other lifestyle modifications that promote symptom relief √ By fostering collaboration between healthcare professionals and people living with tinnitus, we can aim to achieve better symptom management and ultimately improve overall quality of life
Chen HL. et al., 2022 [7]	Taiwan	A comprehensive summary of current possible strategies for the prevention of age-related audiovestibular dysfunction	-	√ A high-fat diet can cause oxidative stress, and low protein intake has been linked to hearing discomfort in the elderly √ Increased carbohydrate and sugar intake positively correlates with the prevalence of audiovestibular dysfunction, while a Mediterranean-style diet may protect against disease. √ Antioxidants in the form of vitamins A, C, and E; physical activity; good sleep quality; quitting smoking; moderate alcohol consumption; and avoiding exposure to noise are also beneficial.
Hofmeister M. et al., 2019 [8]	Germany	A review of the available literature on the effectiveness of a healthy diet, the use of dietary supplements, caffeine restriction, and salt restriction against tinnitus.	-	√ There is very weak evidence that diet quality affects tinnitus symptoms, and further high-quality analytical studies are needed. √ Dietary supplements are ineffective in reducing the symptoms of people with tinnitus and should therefore not be recommended by clinicians. √ There is also no empirical scientific evidence for the commonly recommended restriction of caffeine and dietary salt for patients with tinnitus.
Ismail AMA. et al., 2025 [11]	Egypt	To evaluate the effectiveness of a 12-week intervention involving a calorie-restricted diet and treadmill walking training in older adults with metabolic syndrome (MS) and chronic subjective tinnitus (CSTC).	30 elderly people with metabolic syndrome (MS) and chronic subjective tinnitus (CSTC)	√ BMI, CSTC severity, MS components (WBC, FGIs, blood pressure, Triglycerides, and HDL) and CSTC-related discomfort showed significant improvement in response to a lifestyle-modifying approach in elderly MS with CSTC
Özbey-Yücel Ü. et al., 2021 [12]	Turkey	Assessing the impact of weight loss through diet and physical activity on tinnitus.	46 obese people diagnosed with tinnitus	√ Dietary intervention and physical activity improved tinnitus severity and quality of life in obese tinnitus patients.
Zhang M. et al., 2025 [13]	China	Investigating the relationship between tinnitus prevalence and daily eating patterns.	-	√ Consumption of fruit, dietary fiber, caffeine, and dairy products was negatively correlated with the incidence of tinnitus
Özbey-Yücel Ü. et al., 2023 [21]	Turkey	Determining the impact of nutritional interventions and physical activity on tinnitus symptoms.	63 obese people with tinnitus aged 20 to 65 years	√ Nutritional and physical activity interventions, alone or in combination, have alleviated tinnitus symptoms and increased quality of life in people with tinnitus. √ Organizing nutrition and physical activity programs for obese people with tinnitus would improve the quality of life of these people.

**Table 4:** Potential mechanisms of dietary interventions in MD and tinnitus.

Dietary Intervention	Proposed Mechanism in Ménière’s Disease (MD)	Proposed Mechanism in Tinnitus	Strength of Evidence
Sodium restriction	May reduce fluctuations in endolymph volume and pressure; mechanism based on the classic endolymphatic hypothesis (“the impaired volume and pressure of the endolymph”).	No evidence of benefit; not supported in tinnitus studies.	Low—inconsistent findings; widely used clinically but not confirmed.
Adequate hydration	May stabilize water–electrolyte balance; one study reported improved hearing and reduced vertigo.	No direct evidence of effect.	Low—limited data.
Caffeine reduction	No clear evidence; used as part of general lifestyle modification.	May influence neuronal excitability; individual variability; abrupt withdrawal may worsen symptoms.	Very low—no clinical confirmation.
Alcohol reduction	Possible effect on microcirculation and electrolyte balance; findings inconsistent.	May exacerbate tinnitus perception in some individuals; no consistent evidence.	Very low—observational data only.
Glycemic stabilization/reduced monosaccharides	Possible effect on osmotic pressure of inner ear fluids; evidence limited.	High sugar intake associated with audio-vestibular dysfunction; mechanisms linked to oxidative stress and glucose metabolism.	Low—observational associations.
Anti-inflammatory/Mediterranean/DASH diet	Potential influence on microcirculation and inflammatory activity; indirect evidence.	May reduce oxidative stress and improve endothelial function; supported by population studies.	Low—no interventional trials.
Weight reduction	No direct evidence for MD.	Several studies show improvement in tinnitus severity and quality of life in obese individuals.	Moderate for tinnitus; no data for MD.
Physical activity	No evidence for MD.	Improves tinnitus symptoms when combined with dietary intervention.	Moderate for tinnitus.
Micronutrients (B12, Mg, Zn, vitamin D)	No evidence of association with MD.	Deficiencies may correlate with symptom severity; findings inconsistent.	Very low—heterogeneous results.
High intake of fruit, fiber, dairy; moderate caffeine	No evidence for MD.	Population studies show lower tinnitus prevalence with higher intake of these foods.	Low—observational data

## Data Availability

The original contributions presented in this study are included in the article. Further inquiries can be directed to the corresponding author.

## Abbreviations

The following abbreviations are used in this manuscript:

PCC	Population–Concept–Context
MD	Ménière’s disease
DASH	Dietary Approaches to Stop Hypertension
PRISMA-ScR	Reporting Systematic Reviews and Meta-Analyses
CSTC	Chronic subjective tinnitus
MS	Metabolic syndrome
BMI	Body Mass Index
WBC	White Blood Cell
FGIs	Food Group Indicators
MSG	Monosodium glutamate
